# A pharmacokinetic evaluation and metabolite identification of the GHB receptor antagonist NCS‐382 in mouse informs novel therapeutic strategies for the treatment of GHB intoxication

**DOI:** 10.1002/prp2.265

**Published:** 2016-10-18

**Authors:** Garrett R. Ainslie, K. Michael Gibson, Kara R. Vogel

**Affiliations:** ^1^ Division of Experimental and Systems PharmacologyCollege of PharmacyWashington State UniversitySpokaneWashington

**Keywords:** Diclofenac competition, gamma‐aminobutyric acid, gamma‐hydroxybutyric acid, GABA metabolism, GHB intoxication, GHB receptor antagonism, microsomal metabolism, mouse pharmacokinetics, NCS‐382 dehydrogenation, NCS‐382 glucuronidation, NCS‐382 metabolism, pharmacokinetics, SSADH deficiency

## Abstract

Gamma‐aminobutyric acid (GABA) is an endogenous inhibitory neurotransmitter and precursor of gamma‐hydroxybutyric acid (GHB). NCS‐382 (6,7,8,9‐tetrahydro‐5‐hydroxy‐5H‐benzo‐cyclohept‐6‐ylideneacetic acid), a known GHB receptor antagonist, has shown significant efficacy in a murine model of succinic semialdehyde dehydrogenase deficiency (SSADHD), a heritable neurological disorder featuring chronic elevation of GHB that blocks the final step of GABA degradation. NCS‐382 exposures and elimination pathways remain unknown; therefore, the goal of the present work was to obtain in vivo pharmacokinetic data in a murine model and to identify the NCS‐382 metabolites formed by mouse and human. NCS‐382 single‐dose mouse pharmacokinetics were established following an intraperitoneal injection (100, 300, and 500 mg/kg body weight) and metabolite identification was conducted using HPLC‐MS/MS. Kinetic enzyme assays employed mouse and human liver microsomes. Upon gaining an understanding of the NCS‐382 clearance mechanisms, a chemical inhibitor was used to increase NCS‐382 brain exposure in a pharmacokinetic/pharmacodynamic study. Two major metabolic pathways of NCS‐382 were identified as dehydrogenation and glucuronidation. The *K*_m_ for the dehydrogenation pathway was determined in mouse (*K*_m_ = 29.5 ± 10.0 *μ*mol/L) and human (*K*_m_ = 12.7 ± 4.8 *μ*mol/L) liver microsomes. Comparable parameters for glucuronidation were >100 *μ*mol/L in both species. Inhibition of NCS‐382 glucuronidation, in vivo, by diclofenac resulted in increased NCS‐382 brain concentrations and protective effects in gamma‐butyrolactone‐treated mice. These initial evaluations of NCS‐382 pharmacokinetics and metabolism inform the development of NCS‐382 as a potential therapy for conditions of GHB elevation (including acute intoxication & SSADHD).

**Abbreviations**
ANB: 2‐amino‐2‐norbornane‐carboxylic acid
AUC: area under the concentration–time curve
CL_h_: scaled hepatic clearance
Cl_int,inc_: incubation intrinsic clearance
GABA: gamma‐aminobutyric acid
GBL: gamma‐butyrolactone
GHB: gamma‐hydroxybutyric acid
HLMs: human liver microsomes
K_i_: inhibition constant
MCT1: monocarboxylate transporter 1
MLMs: mouse liver microsomes
NADPH: nicotinamide adenine dinucleotide phosphate
NCS‐382: 6,7,8,9‐tetrahydro‐5‐hydroxy‐5H‐benzo‐cyclohept‐6‐ylideneacetic acid
NL: neutral loss
PBS: phosphate‐buffered saline
SSADHD: succinic semialdehyde dehydrogenase deficiency
UDPGA: uridine 5′‐diphospho‐glucuronosyltransferase

## Introduction

Gamma‐hydroxybutyrate (4‐hydroxybutyrate; GHB), a derivative of the inhibitory neurotransmitter GABA (4‐aminobutyrate; gamma‐aminobutyric acid), is a central nervous system neuromodulator with a storied history. Originally synthesized in the 1960s in the French pharmaceutical industry (Laborit et al. 1960), it was developed as a GABA analog that would cross the blood–brain barrier with interconversion to GABA and concomitant induction of sedation. Safety studies in animals, however, indicated neurotoxicity (absence seizures, bizarre posturing; Snead 1978a,b,c,d). Following a more than decade‐long lacuna, GHB resurfaced in the body‐building population as an agent that purportedly induced growth hormone release via interconversion to GABA (Powers 2012). These individuals (consuming teaspoon quantities of GHB that was, at that time, over the counter) were the first to present to the emergency room with significant respiratory depression and occasionally coma (James 1996; Viera and Yates 1999). Reports of illicit consumption of GHB continued with its purported wide‐scale use in the “rave” club scene as well as its application in sexual assault, the latter associated with its short‐term amnestic effects (Carter et al. 2009a). Today, GHB is employed in multiple settings: (1) as a drug of abuse related to its euphoric properties (schedule I substance); (2) as an FDA‐approved treatment for narcolepsy (sodium oxybate, Xyrem^®^; Jazz Pharmaceuticals, Dublin, Ireland); (3) as a therapeutic intervention for alcoholism in Italy and Austria; (4) as an exploratory therapy for fibromyalgia (www.clinicaltrials.gov); and (5) as an agent to induce absence seizures in rodents (Wong et al. 2004; Snead and Gibson 2005).

**Figure 1:**
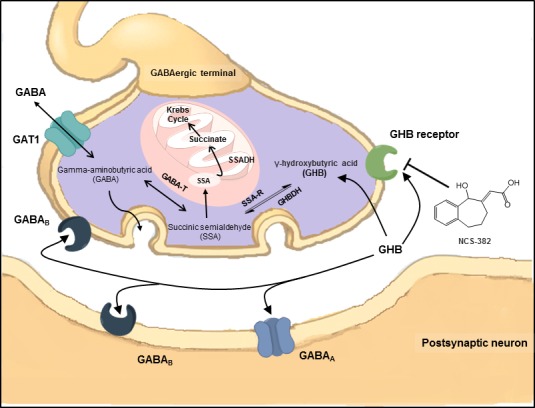
GABA metabolism and the proposed mechanism of NCS‐382. GABA is an important inhibitory neurotransmitter. In the GABAergic terminal, GABA undergoes GABA transaminase‐mediated deamination to form SSA. SSA is taken into the mitochondria where it is further detoxified by succinic semialdehyde dehydrogenase to succinate, prior to entering the Kreb's cycle. SSA can also be converted to GHB, a GHB and GABA_B_ receptor agonist. GHB can activate GABA_B_ receptors located on either the GABAergic presynaptic terminals or the postsynaptic neuron. GHB also activates the GHB receptor. NCS‐382 is a potent GHB receptor antagonist, and prevents GHB binding. GABA, gamma‐aminobutyric acid; GHB, gamma‐hydroxybutyric acid; SSA, succinic semialdehyde.

In addition to GHB intoxication in the illicit setting, a population of patients with inherited deficiency of the GABA‐product metabolizing enzyme, aldehyde dehydrogenase 5a1 (ALDH5A1; also succinic semialdehyde dehydrogenase, SSADH), accumulate supraphysiological levels of both GABA and GHB. This unusual metabolic disorder is the most prevalent heritable defect in the GABA pathway (detailed in Fig. 1), and one for which therapeutic options remain limited. In relation to this, GHBergic receptors exist in the CNS, but their exact structural characteristics and localization remain uncertain (Carter et al. 2009b). GHB exists in the normal mammalian CNS at about 1% of its parent, GABA, but in patients with SSADHD this level is considerably elevated (Malaspina et al. 2016). A major focus of our laboratory is the identification of targeted therapeutics for SSADHD, based on studies in the corresponding knockout mouse (aldh5a1^*−/−*^ mice). Thus far, clinical trials have examined taurine and the GABA_B_ receptor antagonist SGS‐742 (ongoing; www.clinicaltrials.gov). However, no attempt has been made to treat SSADHD with a GHB receptor antagonist despite promising data in the murine model (Gupta et al. 2002).

The specific GHB receptor antagonist, NCS‐382, is a rigid GHB derivative with a *K*_i_ for the GHB receptor some 14 times that of GHB, yet it has never been clinically investigated (Maitre et al. 1990). NCS‐382 has been administered in rodent models as a tool to interrogate GHB function in vivo, albeit in the absence of drug exposure, distribution, and elimination knowledge (Table S1). Accordingly, our goal was to (1) determine murine pharmacokinetics and (2) identify key metabolites and key mechanisms of metabolism to pave the way for a pilot clinical evaluation of NCS‐382 in patients with SSADHD. Eventual clinical development of this neuroactive compound may lead to expanded use in neurological disorders featuring elevated GHB. We have undertaken the first NCS‐382 pharmacokinetic evaluation and initial examination of key metabolites of this agent in mouse‐ and human‐derived enzyme systems. A key mechanism of metabolism was identified in a drug interaction study that increased NCS‐382 efficacy in vivo.

## Materials and methods

### In vivo studies

#### Animals

All animal care and experimental procedures complied with the NIH Guidelines for the Care and Use of Laboratory Animals and were approved by the Washington State University Institutional Animal Care and Use Committee (protocol ASF 04232). Every effort was taken to reduce animal pain and suffering. Animal studies reported are in compliance with the ARRIVE guidelines (McGrath and Lilley 2015). All mice were housed in groups ranging from 1 to 4 animals in a well‐ventilated, temperature‐ and humidity‐controlled room. The animal room was maintained on a 12‐h light–dark cycle (lights on at 7:30 am). Mice had access to food and water ad libitum. This manuscript describes four independent animal studies conducted in mice: (1) an escalating dose drug excretion study, (2) an escalating dose sera and tissue pharmacokinetics study, (3) a drug–drug interaction study, (4) and a pharmacodynamic study.

#### In vivo study 1: Excretion of NCS‐382 in urine and feces

To assess additional routes of NCS‐382 elimination, an independent, nonterminal study was conducted at each dose level (100, 300, and 500 mg/kg) (*n* = 3 mice per group) employing metabolic cages (Braintree Scientific; Braintree, MA) to facilitate the collection of urine and feces. To ensure sufficient volumes of urine could be collected in each of the 2 h time intervals (baseline, 0–2, 2–4, 4–6, 6–8 h), three mice were dosed simultaneously and their urine and feces pooled for analysis. The total dose in *μ*g administered to the three animals was therefore used to calculate the percent of the dose excreted in the urine pooled from all three mice. Animals were allowed ad libitum access to food and water of which the intake of each was monitored. Although this was a nonterminal procedure, any animal receiving an NCS‐382 treatment was not reused for any studies described within.

#### In vivo study 2: Sera and tissue pharmacokinetic studies

C57/B6 mice of mixed gender, aged 4–8 weeks, were bred in house assigned to groups to be administered NCS‐382 (from a 300 mg/mL stock in sterile 0.1% sodium bicarbonate) (100, 300, or 500 mg/kg body weight, i.p.) or vehicle (time zero). In pharmacokinetic studies, mice were not randomized to groups but instead assigned to treatment and time points such that there was no gender of weight bias. Studies were initiated in the morning and following treatment mice were euthanized by CO_2_ asphyxiation and cervical dislocation at predetermined time points (0.25, 0.5, 1, 2, 4, 8, 12, and 24 h) (*n* = 3–4 animals per time point). Blood (via cardiac puncture), brain, liver, and kidney were rapidly collected, tissues were snap‐frozen in liquid nitrogen, and stored at −80°C prior to further analysis. Sera (20–100 *μ*L) was obtained by low‐speed centrifugation at 2000 × g for 10 min at 6°C. Tissues were suspended in phosphate‐buffered saline (PBS, 1:5, w/v) and homogenized via mechanical homogenization before preparation for LC‐MS/MS analysis.

#### In vivo study 3: Inhibition of NCS‐382 glucuronidation

To interrogate the importance of glucuronidation in NCS‐382 elimination in mice, and possibly humans, an interaction study with diclofenac, a potent inhibitor of UDP glucuronosyltransferases (*K*_i_, UGT1A9 = 11 *μ*mol/L and *K*_i_, UGT2B7 = 9 *μ*mol/L) (Miners et al. 1997; Ammon et al. 2000; King et al. 2001), was conducted in mice. In this study, mice (*n* = 3–4 per group) were assigned (balanced for weight and gender) to one of three treatments and collected at one of three time points (0.5, 1, or 2 h). Treatment group 1 received NCS‐382 (300 mg/kg in 0.1% sodium bicarbonate) immediately preceded by 0.1% sodium bicarbonate (vehicle); group 2 received diclofenac (25 mg/kg in sodium bicarbonate) immediately followed by vehicle; and treatment group 3 received diclofenac (25 mg/kg) immediately followed by NCS‐382 (300 mg/kg). Mice were euthanized at the predetermined time points, sera and brain tissue were harvested and stored as described above, and then quantified for NCS‐382, diclofenac, and their metabolites as described next and referenced elsewhere (Sparidans et al. 2008).

#### In vivo study 4: GBL‐mediated response protection by NCS‐382 in the absence and presence of diclofenac

**Figure 2:**
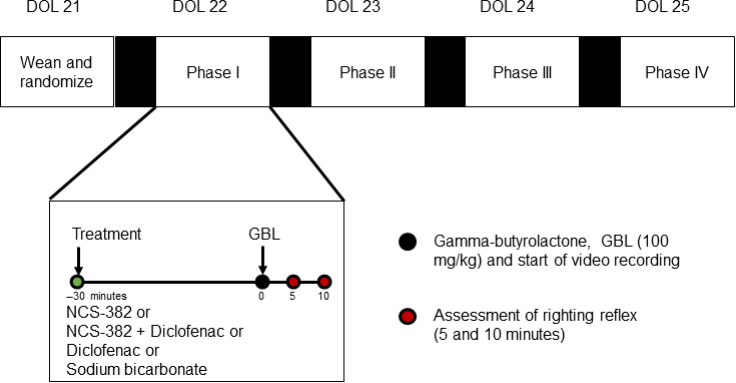
GBL protection study design and procedures. Wild‐type C57/B6 mice (mixed gender, *n* = 7) were bred in‐house, weaned at day of life (DOL) 21, and randomized to a treatment sequence. On each study day, mice received an i.p. injection of either 0.1% sodium bicarbonate (vehicle), NCS‐382 (300 mg/mL), diclofenac (25 mg/mL), or diclofenac and NCS‐382 (administered individually, in that order). Thirty minutes later, mice were given an i.p. injection of GBL (100 mg/kg in PBS) and were placed into a clean round arena (~10 inches in diameter) and were allowed access to food and water. Mice were recorded on a camera operated by Sirena acquisition software (recorded for 10 min). At 5 and 10 min following GBL, righting reflex was assessed. These procedures were repeated daily with ~24 h in between sessions. Data files names were coded such that video analysis was blinded at the time of data recording. PBS, phosphate‐buffered saline.

Diclofenac was dissolved in 0.1% sodium bicarbonate (25 mg/mL) after vortexing and gentle warming (37°C, 30 sec), and NCS‐382 was dissolved in 0.1% sodium bicarbonate (300 mg/mL). Wild‐type C57/B6 mice (mixed gender, *n* = 7) were bred in‐house, weaned at day of life (DOL) 21, and randomized to a treatment sequence (Fig. 2). Preliminary studies with gamma‐butyrolactone (GBL) indicated that younger mice were more susceptible to GBL and results were more reproducible at the selected dose level (data not shown). On each study day, mice received an i.p. injection of either 0.1% sodium bicarbonate (vehicle), NCS‐382 (300 mg/kg), diclofenac (25 mg/kg), or diclofenac and NCS‐382 (administered individually, in that order). Thirty minutes later, mice were given an i.p. injection of GBL (100 mg/kg diluted in PBS) and were placed into a clean round arena (~10 inches in diameter) and were allowed access to food and water. Mice were recorded on a camera operated by Serena acquisition software (recorded for 10 min). At 5 and 10 min following GBL, righting reflex was assessed. These procedures were repeated daily with ~24 h in between sessions. Data file names were coded such that treatments were blinded at the time of video data analysis. During video data analysis, the time of which noticeable loss of motor function or coordination (e.g., “loss of motor function”) was observed, and the time taken for the subject to enter a dissociative state and fall into an unsupported prone position (e.g., “time to loss of motion”) was monitored. Righting reflex was evaluated by placing the mouse in a supine position and the time to return to all four paws was recorded based on the timestamp of the playback video. Data for each treatment group were expressed as the mean and standard deviation, and statistical significance was evaluated using one‐way ANOVA and Tukey post hoc *t* test (*P* < 0.05).

### Quantification of NCS‐328 in mouse serum and tissues by UPLC‐MS/MS

Samples (3 *μ*L) were injected onto a Kinetex PFP (50 × 2.1 mm, 1.7 *μ*m particle size) UPLC column and eluted with a binary gradient consisting of water with 0.1% formic acid (mobile phase A) and methanol with 0.1% formic acid (mobile phase B). Initially, 5% mobile phase B was held for 0.4 min at a flow rate of 0.3 mL/min, then increased linearly to 85% over 2.6 min. This composition was held constant for 0.5 min then returned to initial conditions within 6 sec and equilibrated for an additional 1.8 min. Eluent was directed to waste for the initial 0.4 min then to an ABI Sciex 6500 Qtrap tandem mass spectrometer. Analytes were detected in negative ion mode using multiple reaction monitoring (MRM) transitions for NCS‐382 (216.9→173.1 *m/z*) and ANB (2‐amino‐2‐norbornane‐carboxylic acid, internal standard; 153.9→108.1). The lower limit of quantification (LLOQ) was 14 nmol/L in sera, 60 nmol/g tissue in brain, 60 nmol/g tissue in liver, 10 nmol/g tissue in kidney, 15 *μ*g/mL in urine, and 1.4 *μ*g per 30 mg feces for NCS‐382. Where necessary, samples were diluted in blank matrices for analysis. Methods were developed using the criteria presented in the FDA bioanalytical guidance document (www.fda.gov, 2013).

### Pharmacokinetic data analysis

Pharmacokinetic outcomes were obtained via standard noncompartmental methods using Kinetica (version 5.1, Thermo Fisher Scientific, Waltham, MA). The maximum concentration (*C*_max_) was obtained directly from the concentration–time profiles. Area under the concentration–time curve (AUC) from time zero to the last collected time point was determined using the trapezoidal method with linear up/log down interpolation. For sera and urine, data above the limit of detection but below the LLOQ were computed as LLOQ/2 (7 nmol/L, 7.5 *μ*g/mL, respectively) to allow for the recovery of the terminal half‐life as reported previously (Beal 2001). To obtain estimates of variability around the mean of AUC and *C*_max_, the method of Bailer–Satterwaithe was employed due to the destructive sampling approach (Nedelman et al. 1995). Furthermore, as a result of the destructive sampling approach outcomes for half‐life, apparent volume of distribution and apparent clearance could not be reported with a term for uncertainty.

In the diclofenac–NCS‐382 interaction study, AUC was determined from the data as described above and reported as the interval from 0 to 2 h (AUC_0–2 h_). Additional pharmacokinetic outcomes were not determined in this study.

### Conditions for metabolite identification

Metabolite identification was conducted on samples obtained from pharmacokinetic studies in mice. Analytical conditions and equipment were similar to those described above with some modifications. Samples (10 *μ*L) were injected onto a Kinetex PFP (50 × 2.1 mm, 1.7 *μ*m particle size) UPLC column maintained at 40°C. Mobile phases A and B were identical to those described above; however, the initial composition of 5% mobile phase B was held for 0.4 min, then increased to 85% over 22.6 min, held constant for an additional 3 min, before returning to initial conditions for a total run time of 30 min. MS experiments consisted of enhanced MS (EMS), neutral loss (NL), precursor ion scan (Prec), and MRM each followed by MS/MS experiments when necessary. Constant conditions such as a source temperature (450°C), signal threshold (500 cps), and CAD gas (“high”) were used, while additional parameters (e.g., collision energy, collision energy spread, mass range, etc.) were obtained by the software during data‐dependent scans. Studies were performed using sera and brain samples, with time points pooled for individual matrices and a reference control derived by pooling the sera or brain homogenates from three vehicle‐treated animals.

### In vitro studies

#### Semiquantitative analysis of NCS‐382 metabolites in mouse samples and microsomes

MRM methods for NCS‐382 metabolites were developed using parent and product ion combinations observed in metabolite identification studies and optimized by repeated injection of the same sample, changing mass spectrometer conditions one parameter at a time, to achieve optimal collision energy, source temperature, and source voltages under the chromatographic conditions used for NCS‐382 quantification. These conditions are further described in the results section.

#### NCS‐382 depletion in mouse and human liver microsomes

Incubation mixtures consisted of either mouse liver microsomes (MLMs; pool of male donors) or human liver microsomes (HLMs; pool of 50 mixed gender donors) (0.25 mg/mL), 10 *μ*mol/L NCS‐382 (dissolved in DMSO, <0.06%, and PBS, pH = 7.4). Mixtures (0.1 mL) were preincubated for 5 min at 37°C. Reactions were initiated with the addition of NADPH and UDPGA dissolved in PBS (final concentration of 1 mmol/L and 3 mmol/L, respectively). At predetermined time points, 50 *μ*L aliquots were removed and precipitated using 200 *μ*L of methanol containing 0.1% formic acid and 5 *μ*g/mL ANB. Negative controls were devoid of either protein source or NADPH. All incubations were conducted in triplicate.

#### Determination of in vitro intrinsic clearance

A linear regression model was fit to the data using Graphpad Prism (v6.01; La Jolla, CA). The intrinsic clearance was determined using equation (1).

(1) $$Cl_{\mathrm{int},\mathrm{inc}}=\frac{-k\left(\frac{V}{M}\right)}{f_{u,\mathrm{inc}}}$$

where *k* is the slope, *V* is the volume of the incubation (100 *μ*L), *M* is the amount of microsomal protein in the incubation (0.025 mg), and *f*_u,inc_ is the unbound fraction in the incubation (assumed to equal 1). In vitro intrinsic clearance was scaled to the in vivo intrinsic clearance (Cl'_int_) via equation (2).

(2) $$CL_{\mathrm{int}}^{\prime }=Cl_{\mathrm{int},\mathrm{inc}}*\mathrm{MPPGL}*\mathrm{liver}\;\mathrm{weight}$$

where MPPGL denotes the scaling factor corresponding to the milligrams of microsomal protein per gram of liver (45 mg/g for mouse and 32 mg/g for human), and liver weight is expressed in g/kg of body weight (87.5 g/kg body weight for mouse and 25.7 g/kg body weight for human) (Khojasteh et al. 2011). The hepatic clearance was estimated using the well‐stirred model (eqn. (3)), a free fraction assumed to equal 1, and hepatic blood flow equal to 90 and 32 mL/min/kg body weight for mouse and human, respectively. The well‐stirred model (eqn (3)) was used to estimate whole body clearance for mouse and human.

(3) $$CL_{h}=\frac{Q_{h}*\;f_{u}*Cl_{int}^{\prime }}{Q_{h}+f_{u}*Cl_{\mathrm{int}}^{\prime }}$$

#### Metabolite formation kinetics in MLMs and HLMs

Microsomal incubations were conducted as described above with modifications. NCS‐382 concentrations ranged from 0.1 to 100 *μ*mol/L and the incubation was terminated after 120 min. The *K*_m_ was calculated using nonlinear regression in Graphpad Prism. Due to the lack of authentic standard and relatively short rate of parent disappearance, the maximum formation velocity (*V*_max_) could not be determined for either metabolites.

### Materials

NCS‐382 (6,7,8,9‐tetrahydro‐5‐hydroxy‐5H‐benzo‐cyclohept‐6‐ylideneacetic acid) was obtained from the National Institutes of Health (NIDA). 2‐Amino‐2‐norbornane‐carboxylic acid (ANB), 7‐hydroxycoumarin, diclofenac sodium salt, sodium bicarbonate, nicotinamide adenine dinucleotide phosphate (NADPH), and uridine 5′‐diphospho‐glucuronosyltransferase (UDPGA) were purchased from Sigma‐Aldrich (St. Louis, MO). DMSO, formic acid, methanol, and acetonitrile were purchased from Thermo Fisher (Waltham, MA). Water was obtained from a milli‐Q system (EMD Millipore; Billerica, MA). HLMs and mouse liver microsomes (MLMs) were purchased from Invitrogen (now Thermo Fisher Scientific, Waltham, MA).

## Results

### In vivo study 1: Excretion of NCS‐382 in urine and feces

NCS‐382 low and moderate doses (100 and 300 mg/kg) were selected based on their efficacy in an aldh5a1^−/−^ survival model (Gupta et al. 2002), and the high dose (500 mg/kg) was selected to assess dose linearity for future safety studies. At doses of 100, 300, and 500 mg/kg, 0.1%, 1.1%, and 3.3% of the total dose administered was recovered unchanged in the urine over 8 h, respectively (Fig. S1). Therefore, an increase in NCS‐382 dose resulted in an increased fraction of the dose recovered in the urine. NCS‐382 was detected, but not quantifiable in the feces (<4.5 *μ*g per 30 mg feces).

### In vivo study 2: Sera and tissue pharmacokinetic studies

**Figure 3:**
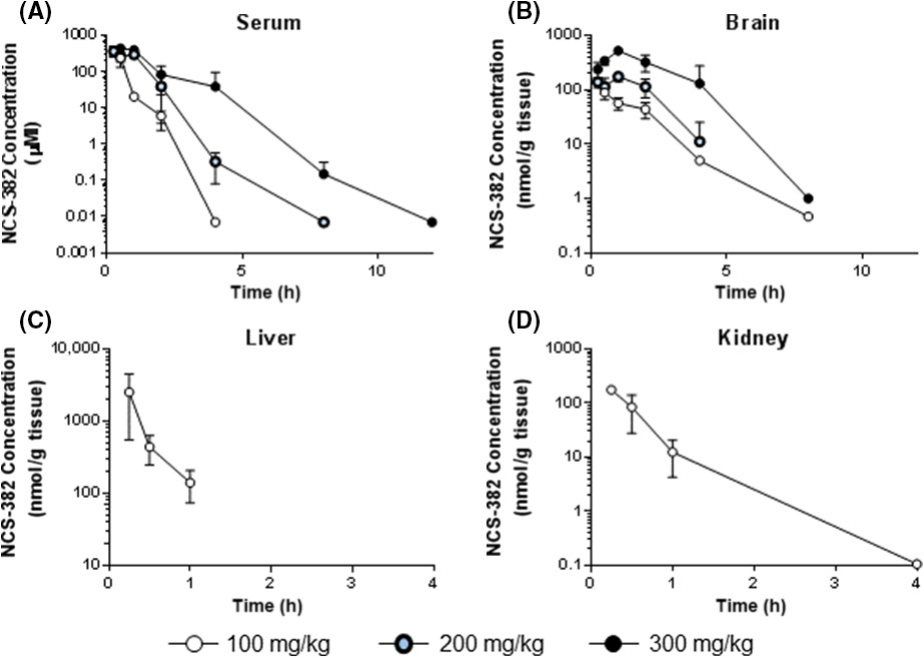
Pharmacokinetic profiles. NCS‐382 concentration–time profiles in sera (A), brain (B), liver (C), and kidney (D) following 100 (open circles), 300 (crimson circles), and 500 (black circles) mg/kg. Data points are presented as the mean (*n* = 3–4 mice per time point), and error bars represent the standard deviation of the mean. Data below the limit of quantification (see methods) are not shown.

**Figure 4:**
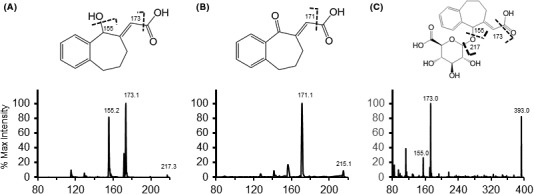
NCS‐382 (A) has a parent ion of 217 [M–H], and is primarily fragmented at the carboxylic acid resulting in an ion at 173, and further fragmentation of the 173 product ion via loss of a water yields an 155 *m/z* ion. NCS‐382 undergoes dehydrogenation (B) revealed by a loss of 2 amu to the parent mass and following loss of the carboxylic acid (173 to 171). Glucuronide conjugation also occurs (C). Glucuronidation (M + 176, 393) was observed to fragment similarly to the parent molecule displaying the most intense signal corresponding to fragmentation of the glucuronide followed by the carboxylic acid.

At the lowest tested dose (100 mg/kg), NCS‐382 was readily absorbed and quantifiable in sera up to 2 h (detectable to 4 h), in liver up to 2 h, in kidney up to 4 h, and in brain up to 8 h after administration of NCS‐382 (Fig. 3). For the 100 mg/kg dose, maximal sera concentrations were fourfold that of brain (Table 1) and >10‐fold that of kidney. The maximal liver concentration was more than 700% increased relative to that of sera. Due to the pharmacological importance of sera and brain levels compared to those in liver or kidney, only sera and brain were measured for NCS‐382 at the higher two doses. With escalating doses (100, 300, and 500 mg/kg), sera and brain exposure were generally dose linear when correcting AUC for dose (Table S1, Fig. 4), although elimination may have approached saturation. Sera terminal half‐life increased with dose, and the apparent clearance (Cl/F) and apparent volume (V/F) were generally consistent. The exception was that of an increase in apparent volume at 500 mg/kg. The terminal half‐life of NCS‐382 was consistently longer in the brain compared to sera for each dose level, but statistics could not be conducted for this outcome, due to the sparse/destructive sampling. Interestingly, an inverse trend between increasing dose was observed with terminal half‐life in the brain. These results coupled with the concentration–time profiles indicate that NCS‐382 may reside preferentially in the liver, following i.p. administration, but remains in the brain and kidney for a longer duration of time (at 100 mg/kg). Brain penetration was improved at the 500 mg/kg dose as indicated by an increase in brain to sera ratio (Table 1). NCS‐382 was undetectable in all samples obtained from vehicle control dosed animals.

**Table 1:** Pharmacokinetic outcomes obtained from noncompartmental analysis

Dose (mg/kg)	100	300	500
Serum			
AUC (*μ*mol/L*h)	119 ± 38	436 ± 42	717 ± 83
C_max_ (*μ*mol/L)	241 ± 106	374 ± 49	451 ± 49
*t*_1/2_ (h)	0.243	0.468	0.683
Cl/F (L/h/kg)	3.8	3.15	3.19
V/F (L/kg)	2.62	2.93	4.25
Brain^a^			
AUC (*μ*mol/L*h)	139 ± 60	313 ± 33	1280 ± 175
C_max_ (*μ*mol/L)	60 ± 9.5	141 ± 45	530 ± 70
*t*_1/2_ (h)	0.967	0.883	0.761
Brain to sera AUC ratio	1.2	0.72	1.8
Liver^b^			
AUC (*μ*mol/L*h)	1150 ± 595	—	—
C_max_ (*μ*mol/L)	1695 ± 2030	—	—
*t*_1/2_ (h)	—	—	—
Kidney^a^			
AUC (*μ*mol/L*h)	24.5 ± 5.3	—	—
C_max_ (*μ*mol/L)	23.6 ± 3.0	—	—
*t*_1/2_ (h)	0.308	—	—

Tissue concentrations assumed equal density to water. AUC, area under the concentration–time curve; *C*_max_, maximal concentration; *t*_max_, time to reach *C*_max_; *t*_1/2_, terminal half‐life; CL/F, apparent clearance; V/F, apparent volume of distribution.

a The concentration reported assumes a tissue density equal to that of water.

b The terminal half‐life could not be determined due to the lack of sufficient (≥3) data points in the elimination phase. Mice (*n* = 3–4 per time point) were administered 100, 300, or 500 mg/kg NCS‐382 dissolved in 0.1% sodium bicarbonate.

During UPLC‐MS/MS analysis for NCS‐382 (retention time, RT = 2.61 min) in mouse sera samples revealed an extraneous peak (RT = 2.48 min, Fig. S2A) that was observed using the MRM transitions for NCS‐382 (216.1→173.1 *m/z*). We hypothesized that this may be due to in‐source fragmentation of an unknown metabolite prompting further investigation of NCS‐382 metabolite formation.

### Initial identification of NCS‐382 metabolites in mouse

NCS‐382‐treated mouse sera and brain samples and their corresponding vehicle‐treated controls were used to identify NCS‐382 metabolites in vivo. Using the Light Sight software, a comprehensive list of prospective phase I and phase II metabolites were targeted (Table S2). Based on enhanced MS scans and predicted metabolite parent *m/z* ratios, NCS‐382 dehydrogenation and glucuronidation products were revealed and could be confirmed via neutral loss and precursor ion scans were conducted. A number of structurally feasible metabolites (including oxidation) were initially identified, but could not be confirmed by their mass fragmentation patterns. High‐resolution mass spectrometry may be required for further inspection of these potential metabolites. MRM methods were developed for the dehydrogenation product and the glucuronide then used for later sample analysis. Uniform declustering potential (−20 V), collision energy (−20 mV), and excitation energy (−13 mV) were determined to be optimal and applied to MRM transitions corresponding to NCS‐382 dehydrogenation (215.1→171 *m/z*) and glucuronidation (393→217 *m/z*). Thorough structural assignment was conducted on NCS‐382, the dehydrogenated metabolite, and the glucuronide (Fig. 4). NCS‐382 ([M‐H] = 217 *m/z*) readily undergoes cleavage of the carboxylic acid resulting in a product ion of 173, subsequent fragmentation and loss of water results in a prominent fragment ion at 155. Dehydrogenation ([M‐H] = 215 *m/z*) fragments in a similar pattern resulting in a product ion of 171 following cleavage of the carboxylic acid, indicating dehydrogenation must occur at the hydroxyl as denoted. Glucuronidation occurs with the addition of 176 ([M‐H + 176] = 393 *m/z*). The loss of the glucuronide results in a product ion equal to the negatively charged parent ion (217 *m/z*) and the characteristic 173 and 155 product ions. Figure 4 denotes one likely site of glucuronidation; mass fragmentation is not sufficient to rule out the formation of the potentially unstable acyl glucuronide. Notably, only the peak eluting at 2.48 min could be detected in vitro. Both peaks were detected in urine samples (Fig. S1B), but not in feces.

### NCS‐382 metabolite formation in MLMs and HLMs

**Figure 5:**
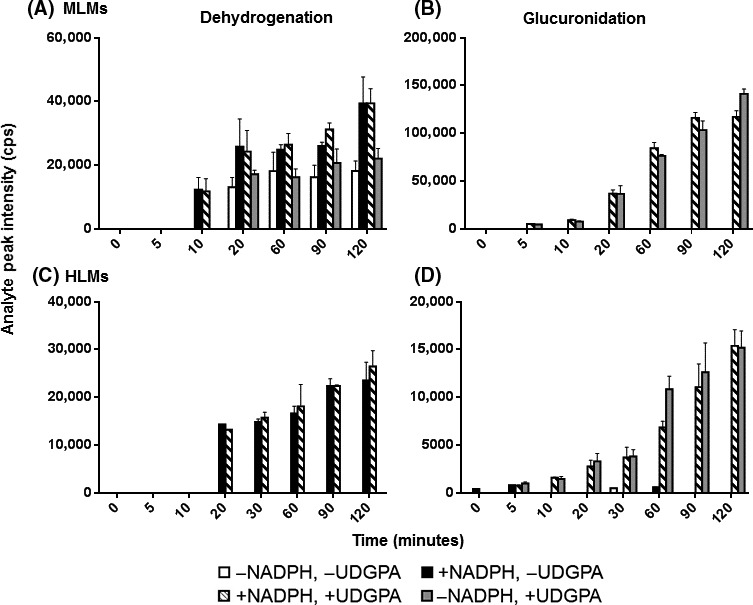
In vitro NCS‐382 metabolism in mouse liver microsomes (Top) and human liver microsomes (bottom). NCS‐382 dehydrogenation of NCS‐382 (left) and glucuronide conjugation (right) in the absence and presence of nicotinamide adenine dinucleotide phosphate (1 mmol/L) and in the absence and presence of uridine 5′‐diphospho‐glucuronosyltransferase (3 mmol/L). Data are presented as the mean ± SD of triplicate incubations.

To examine the in vitro formation of the potential metabolites, incubations of NCS‐382 (100 *μ*mol/L) were conducted in MLMs and HLMs and candidate metabolites were evaluated using MRM mode. In both MLMs and HLMs, the NCS‐382 dehydrogenation product formed over time in the presence of NADPH and NADPH + UDPGA, but not in the absence of NADPH. The NCS‐382 glucuronide conjugate was rapidly formed in the presence of UDPGA regardless of the addition of NADPH (Fig. 5) in either species.

### NCS‐382 clearance in MLMs and HLMs

Linear conditions for protein (0.35 mg/mL) and time (120 min) were established in MLMs and HLMs (Fig. 5, Fig. S3), with <20% of the parent depleted. In vitro intrinsic clearance (Cl_int_) was obtained by NCS‐382 parent disappearance in MLMs (0.587 mL/min/mg protein) and HLMs (0.513 mL/min/mg per protein). Scaling of in vitro data resulted in calculated hepatic clearance of 86.6 mL/min/kg body weight (5.2 L/h/kg body weight) and 20.0 mL/min/kg body weight (1.2 L/h/kg body weight) for mouse and human, respectively.

### Saturable metabolite formation kinetics in HLMs and MLMs

**Figure 6:**
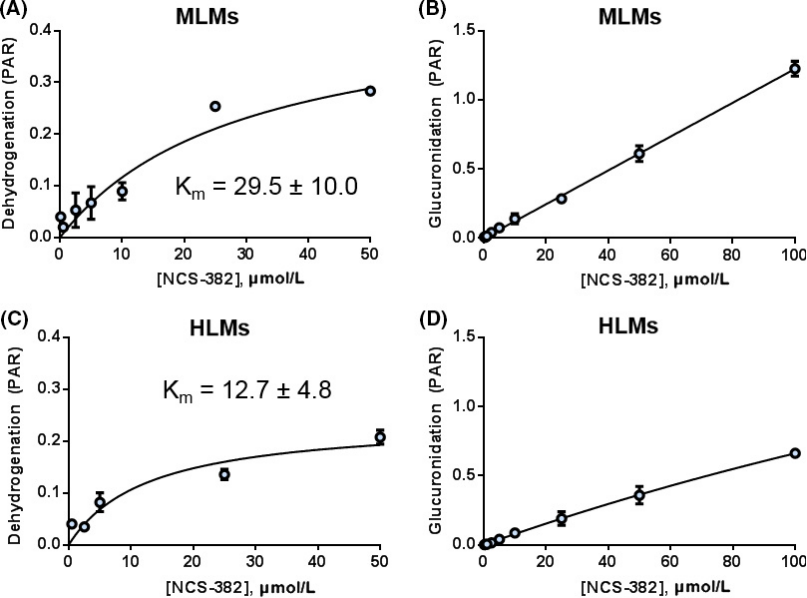
Saturable kinetics of NCS‐382 dehydrogenation (A, C) and glucuronidation (B, D) products in mouse liver microsomes (A, B) and human liver microsomes (C, D). Data are presented as the mean ± SD of triplicate incubations. PAR, analyte peak area to internal standard peak area ratio.

Michaelis constants (*k*_m_) recovered from microsomal incubations were 29.5 ± 10.0 and 12.7 ± 4.8 *μ*mol/L for dehydrogenation in mouse and human liver preparations, respectively (Fig. 6). Due to solubility limitations and the requisite to maintain a low (<1%) fraction of organic solvent in incubations, saturable substrate concentrations were not obtained for glucuronide formation (Fig. 6), although they may be achievable in vivo (Table 1).

### In vivo study 1: Excretion of NCS‐382 metabolites in urine and feces

Following an evaluation of NCS‐382 metabolism both in vivo and in vitro, we reanalyzed samples obtained from study 1, in which urine or feces were collected and quantified for NCS‐382 (Fig. S3). Here, we monitored for the recently identified metabolites. The NCS‐382 glucuronide was detectable in both the urine and feces, but the dehydrogenation product was found in neither (data not shown).

### In vivo study 3: Inhibition of NCS‐382 glucuronidation

**Figure 7:**
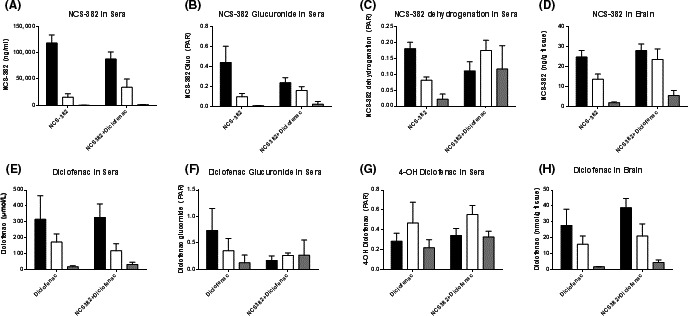
NCS‐382, diclofenac, and metabolite levels in mouse sera and brain. NCS‐382 in sera (A) and brain (D), and diclofenac in sera (E) and brain (H). Glucuronides of NCS‐382 (B) and diclofenac (F) in sera, and NCS‐382 dehydrogenation (C) and diclofenac 4‐hydroxylation products (G) in sera. Data represent the mean ± SD (*n* = 3–4 mice), collected 0.5 (black), 1 (white), or 2 h (gray) post drug administration.

NCS‐382 exhibited exposures consistent with the initial pharmacokinetic study at the same dose (300 mg/kg) and time interval (0–2 h, Table 2). The presence of diclofenac (25 mg/kg), an inhibitor of glucuronidation, did not change the sera AUC_0‐2 h_ of NCS‐382, but it did significantly increase the brain AUC_0–2 h_. NCS‐382 glucuronide formation was decreased in the presence of diclofenac in sera, but not brain (Fig. 7, Table 2). Peak area ratio of the dehydrogenated NCS‐382 species were increased in the diclofenac‐treated mice, relative to NCS‐382 treatment alone.

**Table 2:** Drug and metabolite exposure outcomes in an NCS‐382–diclofenac interaction study

	Treatment, AUC_0–2 h_ [90% CI]		
	NCS‐382	NCS‐382 + Diclofenac	Diclofenac
Sera			
NCS‐382 (*μ*mol/L*h)	411 [362–460]	379 [327–431]	—
Diclofenac (*μ*mol/L*h)	—	218 [193–243]	232 [197–266]
5‐OH‐Diclofenac (*μ*mol/L*h)	—	54.3 [43.3–65.3]	61.3 [48.1–74.6]
NCS‐Glucuronide (PAR*h)^b^	3.65 [2.65–4.65]	2.86 [2.55–3.18]	—
NCS dehydrogenation (PAR*h)^b^	1.99 [1.82–2.16]	2.72 [2.26–3.18]^a^	—
Diclofenac glucuronide (PAR*h)^b^	—	4.67 [4.21–5.13]^a^	8.57 [6.09–11.04]
4‐OH‐Diclofenac (PAR*h)^b^	—	8.28 [7.60–8.97]	6.76 [5.18–8.33]
Brain			
NCS‐382 (nmol/g/tissue*h)	128 [115–141]	182 [164–200]^a^	—
Diclofenac (nmol/g/tissue*h)	—	44.9 [39.5–50.3]^a^	33.8 [28.2–39.4]
5‐OH‐Diclofenac (nmol g/tissue*h)	—	5.20 [4.77–5.62]^a^	4.60 [4.55–4.65]
NCS‐Glucuronide (PAR*h)^b^	3.28 [2.53–4.03]	4.58 [3.61–5.55]	—
Diclofenac glucuronide (PAR*h)^b^	—	3.96 [3.25–4.67]	5.87 [4.46–7.28]
4‐OH‐Diclofenac (PAR*h)^b^	—	15.9 [13.6–18.2]	7.9 [6.9–9.0]

AUC, partial area under the sera concentration–time curve from 0.5 to 2 h; NCS‐382 was administered at 300 mg/kg and diclofenac at 25 mg/kg.

a A significant increase compared to respective control treatment (*P* < 0.05).

b Only relative quantification was conducted, therefore concentration units are expressed as peak area ratio (PAR); hepatic oxidation of diclofenac leads to the major metabolite 4′‐hydroxydiclofenac, 5‐hydroxydiclofenac, as well as 4′,5‐dihydroxydiclofenac. The NCS‐382 dehydrogenation product was undetected in brain homogenates.

Diclofenac sera exposures were unchanged in the presence of NCS‐382 compared to diclofenac treatment alone (Table 2, Fig. 7). Coadministration of these UGT substrates resulted in decreased formation of the diclofenac glucuronide; however, there was no significant change in the levels of 5‐hydroxy‐ and 4‐hydroxy‐diclofenac in sera (Table 2, Fig. 6 and Fig. S3). The 4‐hydroxy‐diclofenac levels in the brain were unchanged, but diclofenac was elevated and its glucuronide was decreased in the presence of NCS‐382. The 5‐hydroxy‐diclofenac exposures were increased in the brain of mice coadministered NCS‐382.

### In vivo study 4: GBL‐mediated response protection by NCS‐382 in the absence and presence of diclofenac

**Figure 8:**
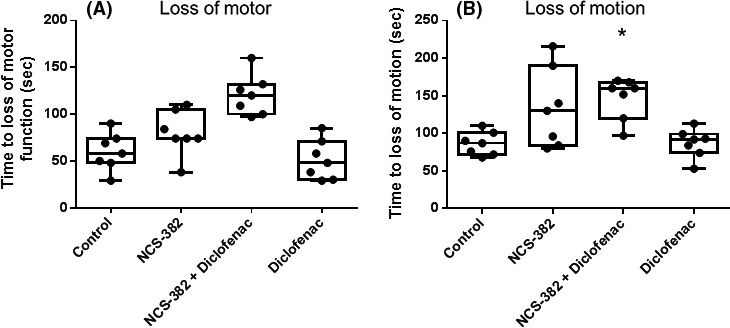
NCS‐382 is protective against GBL‐induced intoxication. Mice were pretreated with either vehicle (black) NCS‐382 (blue), NCS‐382 and diclofenac (blue with hash), or diclofenac (hash) and administered GBL (100 mg/kg) and their time to loss of motor function (A) or entry into a dissociative state (B) was measured. Bar denotes the mean ± SD (*n* = 7 mice).

In order to observe if a diclofenac‐mediated increase in exposure would translate to a measureable pharmacodynamic effect, mice were challenged with GBL (100 mg/kg), a GHB prodrug readily converted to GHB by the action of plasma esterases. In the vehicle pretreatment phase of this study, mice developed evidence of loss of motor function ~1 min after GBL treatment. Mice then entered a disassociated state where they no longer supported themselves with their forelimbs at ~1.5 min. NCS‐382 alone trended toward increasing the time to loss of motor function, while the addition of diclofenac to NCS‐382 significantly improved this metric (Fig. 8). Diclofenac, at the dose tested, in the absence of NCS‐382 did not have a measureable effect compared to vehicle control. Righting reflex was measured but in most cases the time was too short to accurately capture (<1 sec). There were two mice, however, that exhibited longer righting reflex times after 5 min in either the vehicle phase of the NCS‐382 only phase (12–60 sec). Considering this high dose of GBL, protection by NCS‐382 in the presence of diclofenac is considerable, and lower doses (70 mg/kg) given to older and less susceptible mice, have induced behavior changes and 3–6 Hz spike and wave discharge (Ishige et al. 1996).

## Discussion

NCS‐382 was developed as a GHB analog and potent GHB receptor antagonist to elucidate the role of GABA and GHB receptor signaling pathways (Fig. 1). Due to its potent *K*_i_ (1.2 *μ*mol/L) and selectivity (Molnár et al. 2006), NCS‐382 has been proposed as an antidote to GHB intoxication, as well as a potential therapeutic consideration for SSADHD, a condition of chronically elevated GHB (Gupta et al. 2002). NCS‐382 has also been employed as a GHB receptor antagonist in myriad preclinical studies to elucidate GHB receptor function (Table S1), all in the absence of pharmacokinetic information and confirmation that NCS‐382 crosses the blood–brain barrier. In the present work, we sought to address some of the gaps in knowledge of its pharmacokinetics and metabolism.

A 24‐h pharmacokinetic study in which a single i.p. bolus (100 mg/kg) was administered to mice provides a rudimentary understanding of NCS‐382 distribution, kinetics, and metabolism, employing the most common route of NCS‐382 administration in the literature. Increased doses (300 and 500 mg/kg), where sera and brain concentrations were measured, indicated linear kinetics in the range of 100–500 mg/kg, based on sera exposure (Fig. S4A and B), however, terminal half‐life increased with dose in sera and trended to decrease in brain (Fig. S4C). Due to the destructive sampling, the standard deviation for terminal half‐life could not be determined and statistical changes could not be assessed. The absorption of NCS‐382 was rapid regardless of the dosage level, precluding efficient capture of the absorption phase. Since i.p. administered drugs are absorbed via portal circulation (Lukas et al. 1971) and undergo first‐pass effects, only apparent volume and clearance could be recovered. Sera concentrations well above the reported GHB receptor *K*_i_ were achieved. The half‐life of NCS‐382 was short and was almost completely eliminated from sera and liver after only 2–4 h. NCS‐382 had a prolonged residence in the brain with quantifiable levels for at least 8 h following the dose. One explanation is that the high‐affinity binding to GHB receptors in the brain allow for its retention and unperturbed return to systemic circulation. The trend of a decrease in brain terminal half‐life with increasing dose may be the result of saturation of GHB binding receptors resulting in more unbound NCS‐382 for return to systemic circulation. The longer exposure of NCS‐382 in the kidney is less readily explained. One possible explanation involves an alternative mechanism. GHB is a ligand for the monocarboxylate transporter 1 (MCT1) (*K*_m_^ ^= 4.6–7.7 mmol/L) (Tamai et al. 1999; Wang and Morris 2007), present in both the brain and the kidney; for the latter, reabsorption from the urine can occur. Inhibition of the MCT transporter has even been proposed as a means to increase renal clearance for GHB in cases of intoxication (Morse and Morris 2013). NCS‐382 may also be a ligand for the MCT transporter and subsequent competitive inhibition of the transporter may decrease exposure and half‐life. The low percentage of NCS‐382 dose recovered in the urine, increasing with dose, further supports the hypothesis, that NCS‐382 is an MCT substrate, but this should be confirmed experimentally in future work. The increase of the fraction excreted in the urine with increasing dose could also be the result of saturated efflux mechanisms in the liver, or of saturable metabolic clearance undergoing compensation by renal excretion.

Importantly, metabolism was demonstrated as a major elimination pathway for NCS‐382, and therefore the identification of key metabolites was prioritized. The objective of this study was to identify and examine key metabolites, of which structural confirmation could be determined with our present resources. Of those metabolites, the glucuronide conjugate appears to represent a key biotransformation for NCS‐382 in both mouse and human (Fig. 6). To further evaluate the in vivo relevance of the glucuronidation pathway, we employed the UGT2B7 inhibitor, diclofenac, and measured key metabolites of both drugs in mouse sera and brain at 0.5, 1, and 2 h following treatment. As expected, the coadministration of NCS‐382 and diclofenac resulted in a decrease in NCS‐382 glucuronide formation, which appeared to be compensated for by the dehydrogenation pathway, and possibly other unidentified metabolic pathways. Interestingly, the diclofenac glucuronide was formed to a lesser extent in the presence of NCS‐382 and the 4‐ and 5‐hydroxy metabolites were unchanged. There are additional hydroxylated metabolites that we were unable to quantify that may account for the lack of change in diclofenac sera exposure. The increase in NCS‐382 in the brain was accompanied by an increase in diclofenac and 5‐hydroxy diclofenac levels in the brain during coadministration. Diclofenac, and its acyl glucuronide, has been implicated as a substrate and inhibitor of both MRP1‐4 and OATP1A4 (Kawase et al. 2016; Zhang et al. 2016), which warrants further interrogation of NCS‐382 transport mechanisms.

To determine if this increase in NCS‐382 levels in the brain would alter its pharmacological effect, mice were pretreated with either vehicle, NCS‐382, diclofenac, or both NCS‐382 and diclofenac, 30 min prior to GBL. NCS‐382 alone trended toward having a protective effect on the pharmacological metrics which were significantly improved in the presence of diclofenac (Fig. 8), although diclofenac alone did not have a statistically significant protective effect. Diclofenac has been reported to be a ligand for the GHB binding sites in rat brain, but in this pharmacological model did not exert an antagonistic effect (Wellendorph et al. 2009). Due to the alterations of diclofenac exposures during coadministration with NCS‐382, effects of diclofenac metabolites cannot be ruled out as contributors to the increased protective effect against GBL.

## Conclusions

Overall, the in vivo pharmacokinetic and in vitro metabolism studies suggest that NCS‐382 is well absorbed in mouse and eliminated primarily via hepatic metabolism. Two key metabolites were identified corresponding to dehydrogenation and glucuronidation of NCS‐382. Both metabolites were formed via human‐ and mouse‐derived enzyme systems and with comparable interspecies kinetics of formation. Although more exhaustive efforts to elucidate NCS‐382 metabolites, including treatment with radiolabeled compound and detection by high‐resolution mass spectrometry, need to be conducted for clinical development, these data establish glucuronidation as an elimination pathway in both mice and humans. NCS‐382 glucuronidation was successfully inhibited and NCS‐382 brain levels were increased in the presence of diclofenac indicating a role for UGT2B7 in the metabolism of NCS‐382. The increased brain levels corresponded to an improved protective effect against GBL. This coupled to the significant lifespan extension of SSADHD mice by NCS‐382 highlights its potential therapeutic application in conditions of supraphysiological GHB accumulation. Our current findings advance the preclinical development of NCS‐382 as a treatment consideration for GHB intoxication.

## Author Contributions

Ainslie, Vogel, and Gibson participated in the research design and wrote or contributed to the writing of the manuscript. Ainslie and Vogel conducted experiments and performed data analysis. Gibson contributed to new reagents and analytic tools.

## Disclosure

None declared.

## Supporting information


**Figure S1.** NCS‐382 recovered in urine collected from 0 to 8 h post NCS‐382 dose. Each data point represents a single measurement of pooled urine at each collection midpoints (*n* = 3 animals/dose group).

**Figure S2**. UPLC‐MS/MS chromatograms obtained from sera (A) and urine (B), both following an i.p. dose of 300 mg/kg NCS‐382. NCS‐382 (retention time, RT = 2.61 min) and an unknown interference (RT = 2.48 min) were detected in the sera (A). NSC‐382 glucuronide transitions (393.1→217.1 *m/z*) identified two peaks (RT = 2.48 and 2.58 min) in mouse urine collected 0–2 h after administration. Units on the *y*‐axis (cps) denote counts per second.

**Figure S3**. Linearity of NCS‐382 metabolite formation over time. NCS‐382 dehydrogenation (A, C) and glucuronidation (B, D) in MLMs and HLMs, respectively. Data represent the mean ± SD of triplicate incubations.

**Figure S4**. NCS‐382 pharmacokinetic outcomes with increasing dose in sera and brain. NCS‐382 exposure (AUC, A), peak concentration (*C*_max_, B), and terminal half‐life (*t*_1/2_, C) with increasing NCS‐382 dose in brain (white circle) and sera (black circles). Data represent the mean ± SD. Due to sparse sampling techniques a SD could not be determined for *t*_1/2_.

**Figure S5**. NCS‐382 and diclofenac metabolite levels in mouse sera and brain. NCS‐382 glucuronide in sera (A) and brain (D) and 5‐hydroxydiclofenac in sera (E) and brain (H). Glucuronides of NCS‐382 (B) and diclofenac (F) in sera and NCS‐382 dehydrogenation (C) and diclofenac 4‐hydroxylation products (G) in sera. Data represent the mean ± SD (*n* = 3–4 mice), collected 0.5 (black), 1 (white), or 2 h (gray) post drug administration.

**Table S1**. In vivo studies reporting NCS‐382 administration since 1995 and key outcomes.

**Table S2.** Characteristics of candidate NCS‐382 metabolites.
