# Supplementary figures and images for: A pharmacokinetic evaluation and metabolite identification of the GHB receptor antagonist NCS‐382 in mouse informs novel therapeutic strategies for the treatment of GHB intoxication
Source: Pharmacol Res Perspect. 2016 Oct 18;4(6):e00265. doi: 10.1002/prp2.265 (PMC5115179; doi:10.1002/prp2.265)

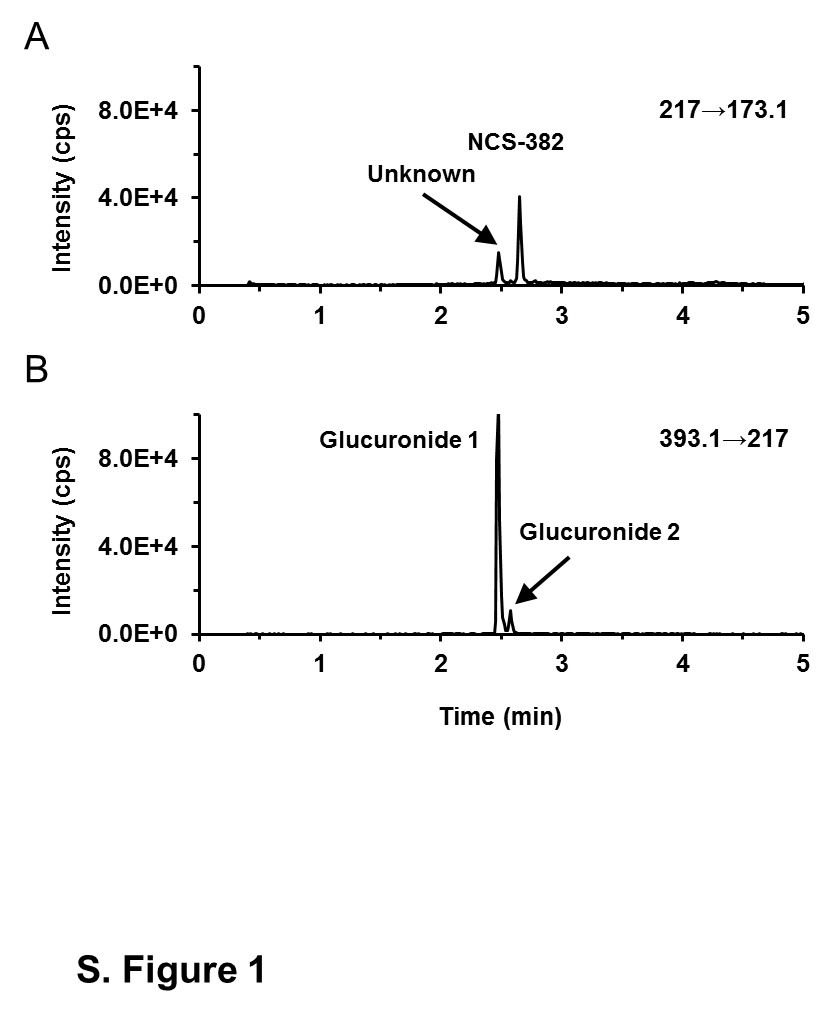

Supplement: Supplementary file 1 — Figure S1. NCS‐382 recovered in urine collected from 0 to 8 h post NCS‐382 dose. Each data point represents a single measurement of pooled urine at each collection midpoints (n = 3 animals/dose group). [file PRP2-4-e00265-s001.jpg]

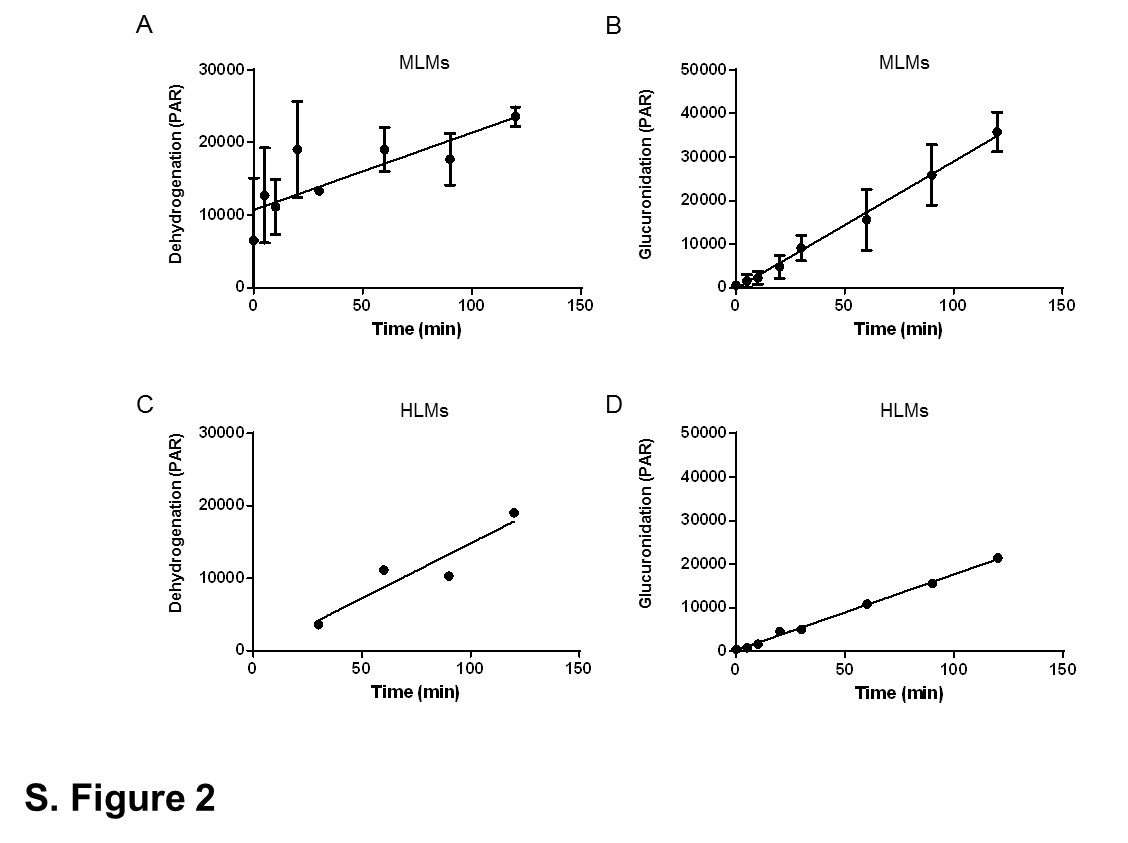

Supplement: Supplementary file 2 — Figure S2. UPLC‐MS/MS chromatograms obtained from sera (A) and urine (B), both following an i.p. dose of 300 mg/kg NCS‐382. NCS‐382 (retention time, RT = 2.61 min) and an unknown interference (RT = 2.48 min) were detected in the sera (A). NSC‐382 glucuronide transitions (393.1→217.1 m/z) identified two peaks (RT = 2.48 and 2.58 min) in mouse urine collected 0–2 h after administration. Units on the y‐axis (cps) denote counts per second. [file PRP2-4-e00265-s002.jpg]

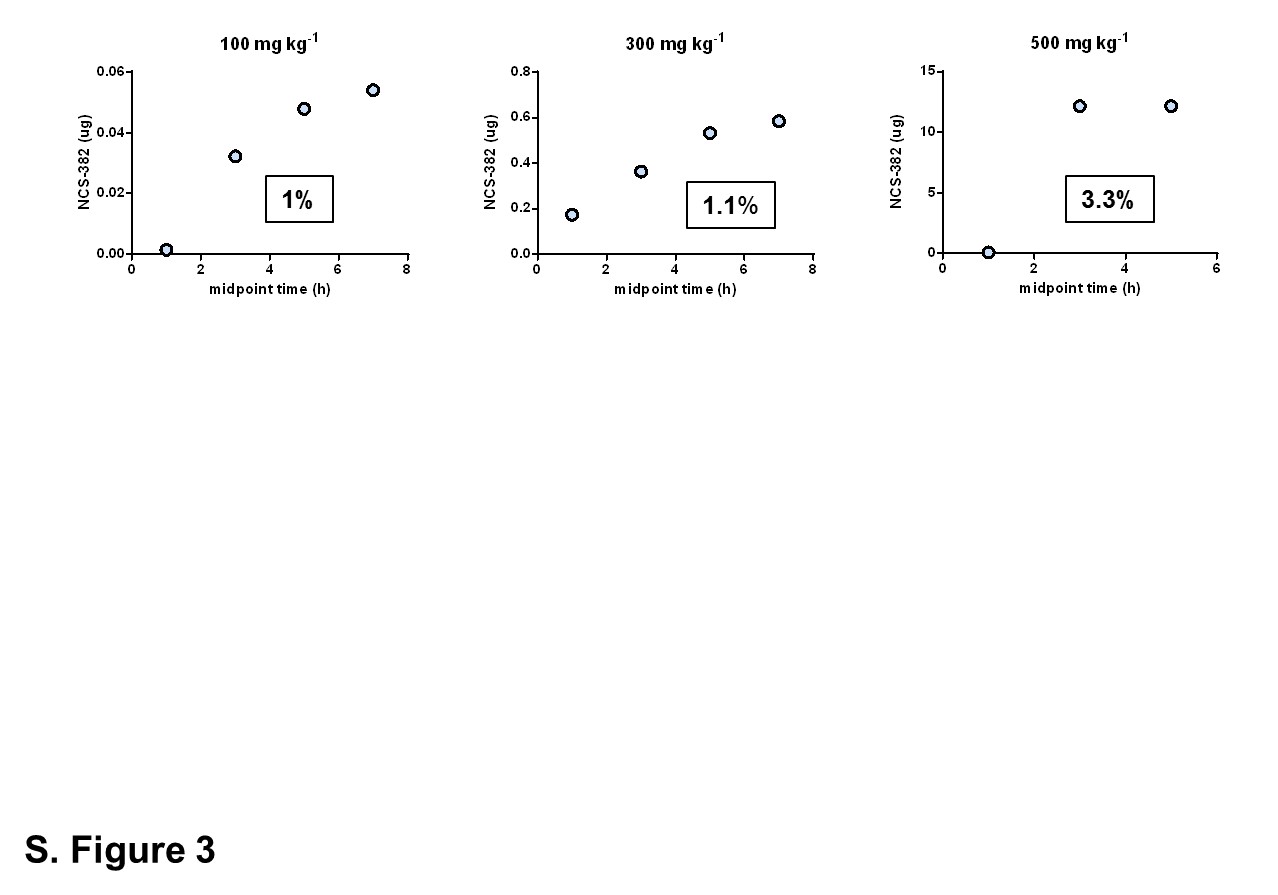

Supplement: Supplementary file 3 — Figure S3. Linearity of NCS‐382 metabolite formation over time. NCS‐382 dehydrogenation (A, C) and glucuronidation (B, D) in MLMs and HLMs, respectively. Data represent the mean ± SD of triplicate incubations. [file PRP2-4-e00265-s003.jpg]

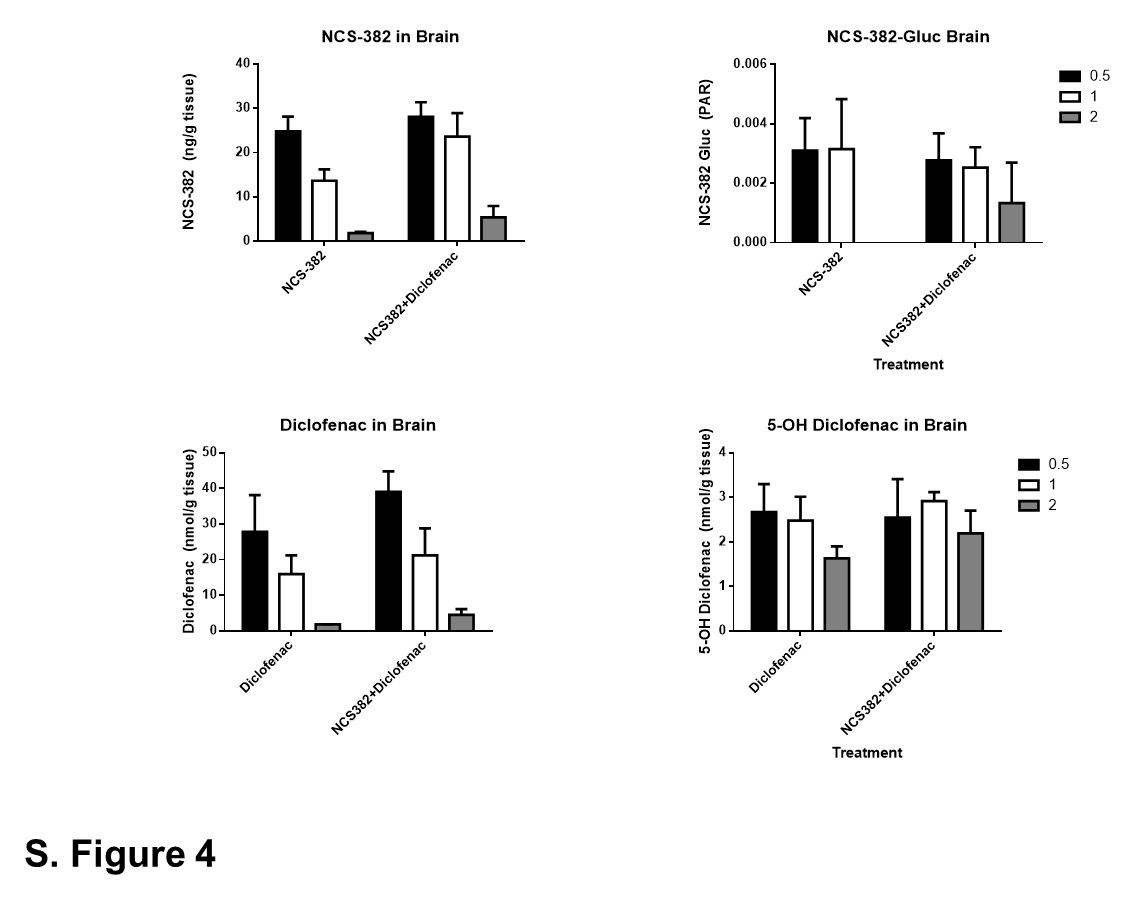

Supplement: Supplementary file 4 — Figure S4. NCS‐382 pharmacokinetic outcomes with increasing dose in sera and brain. NCS‐382 exposure (AUC, A), peak concentration (C max, B), and terminal half‐life (t 1/2, C) with increasing NCS‐382 dose in brain (white circle) and sera (black circles). Data represent the mean ± SD. Due to sparse sampling techniques a SD could not be determined for t 1/2. [file PRP2-4-e00265-s004.jpg]

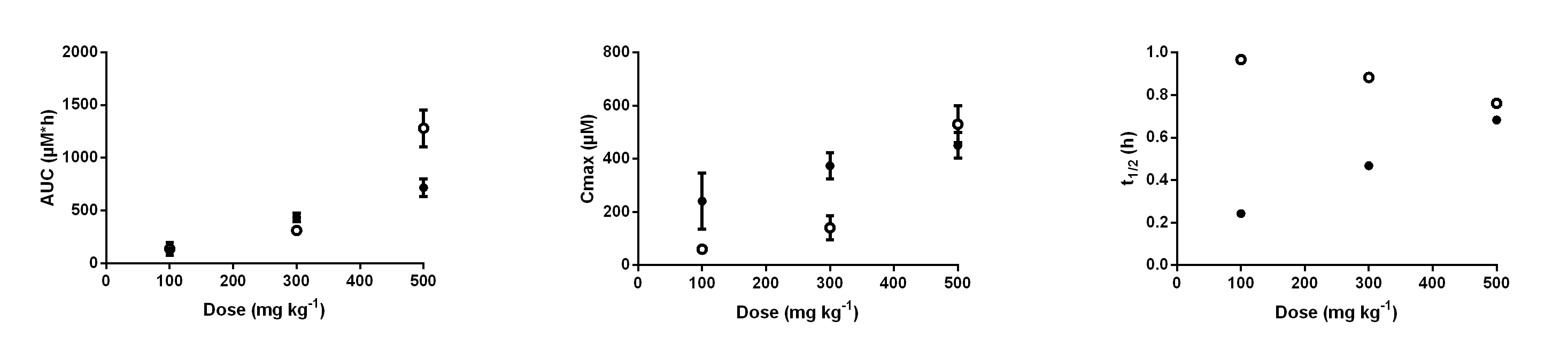

Supplement: Supplementary file 5 — Figure S5. NCS‐382 and diclofenac metabolite levels in mouse sera and brain. NCS‐382 glucuronide in sera (A) and brain (D) and 5‐hydroxydiclofenac in sera (E) and brain (H). Glucuronides of NCS‐382 (B) and diclofenac (F) in sera and NCS‐382 dehydrogenation (C) and diclofenac 4‐hydroxylation products (G) in sera. Data represent the mean ± SD (n = 3–4 mice), collected 0.5 (black), 1 (white), or 2 h (gray) post drug administration. [file PRP2-4-e00265-s005.jpg]
