# Supplementary material for: A pharmacokinetic evaluation and metabolite identification of the GHB receptor antagonist NCS‐382 in mouse informs novel therapeutic strategies for the treatment of GHB intoxication
Source: Pharmacol Res Perspect. 2016 Oct 18;4(6):e00265. doi: 10.1002/prp2.265 (PMC5115179; doi:10.1002/prp2.265)
Supplement: Supplementary file 6 — Table S1. In vivo studies reporting NCS‐382 administration since 1995 and key outcomes. [file PRP2-4-e00265-s006.docx]

**S. Table 1**. In vivo studies reporting NCS-382 administration since 1995 and key outcomes.

| Species | Dose level  mg/kg^a^ | Dose Route | Key Results | Reference |
| --- | --- | --- | --- | --- |
| mouse | 1, 4, 25 ug | i.c.v, i.p. | GHB receptors are important targets for the hypnotic and analgesic effects induced by emulsiﬁed enﬂurane, isoﬂurane, and sevoﬂurane. | (Wang et al., 2014) |
| mouse | 1, 5, 25 ug | i.t. |  |  |
| rat | 50 | i.p. | GHB Fos expression is GHB and GABA_B_R independent; NCS-382 doses were limited. | (van Nieuwenhuijzen et al., 2014) |
| rat | 10 | i.p. | NCS-382 prevents both neurological damage and working- memory impairment induced by low-dose GHB. | (Pedraza et al., 2009) |
| rat | 25, 50, 100 | i.g. | GHB's (300 mg/kg) discriminative stimulus effects were obliterated by the GABA_B_ receptor antagonist, CGP-35348 and slightly attenuated by NCS 382. | (Baker et al., 2008) |
| mouse | 50, 100, 250, 500 | ns | SCH-50911 was protective against GBL-associated lethality; NCS-382 application indicated these effects involved GABA_B_ receptor activation. | (Pedraza et al., 2009) |
| pigeon | 54, 178, 320, 560 | ns | GHB, its precursors 1,4-BD and GBL, and NCS-382 did not attenuate the effects of diazepam. | (Koek et al., 2006) |
| rat | 100 | i.p. | GHB and its metabolic precursors produce similar subjective effects, unique from those of other sedative-hypnotic drugs tested. | (Baker et al., 2005) |
| mouse | 50, 100 | i.p. | GABA_B_ receptors are involved in the lethal effect of GHB in rodents. | (Carai et al., 2005) |
| rat | ns | ns | GHB and baclofen are structurally similar, but pharmacologically differ. These differences may be due to GHB receptors involvement or drug interactions with GABA_B_ receptors. | (Koek et al., 2005) |
| Baboon | 0.1-10 | i.m. | GABA_B_ receptors are important in mediating the behavioral effects of GHB in baboon; GHB receptor involvement remains less clear. | (Goodwin et al., 2005) |
| rat | 50 | i.v. | NCS-382 reversed the ↑ in heart rate but not the pressor response induced by GHB. | (Hicks et al., 2004) |
| rat | 10, 32, 56, 100 | i.p. | GHB and its prodrugs vary in potency and duration of action. CGP-35348 and NCS-382 failed to antagonize the effects of baclofen on motor effects. NCS-382 lack of effect may be due to its own GHB-like effects. | (Carter et al., 2004) |
| mouse | 200 | i.p. | In mouse hippocampus and frontal cortex, GHB reduced mitogen-activated protein kinase phosphorylation by GABA_B_ receptor. | (Ren and Mody, 2003) |
| rat | 3.2, 32 | i.p | NCS-382 did not antagonize the motor effects of GHB, consistent with the belief that many of the behavioral actions of exogenously administered GHB result from GHB's actions at sites other than the GHB receptor. | (Lamb et al., 2003) |
| rat | 10, 32, 56, 100 | i.p. | The effects of 1,4-butanediol under these conditions result from its conversion to GHB. NCS-382 may shares effects with GHB. | (Carter et al., 2003) |
| mouse | 25, 50, 75 | i.p. | ↓ gut motility induced by GHB is the result ot GABA_B_ receptor activation. | (Carai et al., 2002a) |
| mouse | 200 | i.p. | Pretreatment with NCS-382 alone produced prolonged failure of the rotator test, and a synergistic effect with 1,4-BD (GHB prodrug). | (Quang et al., 2002) |
| mouse | 50, 100, 200, 300 | i.p. | Taurine and NCS-382 may have therapeutic relevance in human SSADH deficiency. | (Gupta et al., 2002) |
| mouse | 200 | ns | Sedative/hypnotic effects of 1,4-BD are due to conversion to GHB which binds to GABA_B_ receptors. | (Carai et al., 2002b) |
| rat | 50 | i.p. | GHB, via a GABA_B_ receptor-mediated mechanism, increases the brain concentrations of neurosteroids. | (Barbaccia et al., 2002) |
| rat | n.s. | i.p. | Tissue serotonin turnover can be initiated by GHB and may be due to an ↑ in tryptophan transport to the brain and subsequent uptake by serotonergic cells. | (Gobaille et al., 2002) |
| rat | n.s. | i.p. | GHB-induced ↓ of hippocampal acetylcholine release is mediated by GABA_B_ receptors, based on ineffective blocking by NCS-382. | (Nava et al., 2001) |
| mouse | 50, 100, 250, 500 | i.p. | Sedative/hypnotic effects of GHB are mediated by the activation of GABA_B_ receptors. | (Carai et al., 2001) |
| rat | 10 | i.p. | GHB (at low doses) selectively activates GHB receptors to impact REM sleep. | (Tremblay et al., 1998) |
| rat | 150 | i.p. | Gamma-hydroxybutyric acid is not an effective growth hormone secretagogue. | (Rigamonti and Müller, 2000) |
| rat | 2 mmol/kg | i.p. | GHB modulates the extracellular concentrations of GABA in certain regions of the rat brain. | (Gobaille et al., 1999) |
| rat | 25, 100, 200 | p.o. | NCS-382, dose dependently, ↓ gastric emptying and antagonized the prokinetic effect of GHB. | (Poggioli et al., 1999) |
| rat | 400 | i.p. | NCS-382 reduces blood ethanol levels, likely due to a lessened absorption of ethanol from the gastrointestinal tract. | (Colombo et al., 1999) |
| mouse | 12.5 | i.p. | GHB was shown to have abuse potential, which was blocked by NCS-382. | (Martellotta et al., 1998) |
| rat | 100 | i.v. | GABA_B_ receptor activation by GHB ↓ signal firing rate and alters firing patterns of nigral dopamine neurons. | (Erhardt et al., 1998) |
| rat | 300 | i.p. | The anxiolytic effect of GHB were reversed by the the benzodiazepine receptor antagonist, flumazenil, but not by naloxone or NCS-382. | (Schmidt-Mutter et al., 1998) |
| mouse | 50 | i.p. | Related to the pathogenesis of absence seizures, baclofen and GHB have different effects on long-term potentiation in the CA1 region of the hippocampus despite a common mode of action on the thalamocortical functions. | (Aizawa et al., 1997) |
| rat | 100-500 | i.p. | NCS-382 was protective against two chemically induced models for absence seizures. | (Snead III, 1996) |
| rat | 12.5, 25, 50 | i.p. | Blockade of the discriminative stimulus effects of gamma-hydroxybutyric acid (GHB) by the GHB receptor antagonist NCS-382. | (Colombo et al., 1995) |
|  |  |  |  |  |

Intracerebroventricularly, i.c.v; intrathecally, i.t.; intragastric, i.g.; not specified, ns; intramuscular, im; gamma-butyric acid, GHB; gamma-butyrolactone (GHB prodrug), GBL;

^a^ unless otherwise denoted.

**References**

Aizawa, M., Ito, Y., and Fukuda, H. (1997). Roles of gamma-aminobutyric acidB (GABA B) and gamma-hydroxybutyric acid receptors in hippocampal long-term potentiation and pathogenesis of absence seizures. Biol. Pharm. Bull. *20*: 1066–1070.

Baker, L.E., Searcy, G.D., Pynnonen, D.M., and Poling, A. (2008). Differentiating the discriminative stimulus effects of gamma-hydroxybutyrate and ethanol in a three-choice drug discrimination procedure in rats. Pharmacol. Biochem. Behav. *89*: 598–607.

Baker, L.E., Tilburg, T.J.V., Brandt, A.E., and Poling, A. (2005). Discriminative stimulus effects of gamma-hydroxybutyrate (GHB) and its metabolic precursor, gamma-butyrolactone (GBL) in rats. Psychopharmacology (Berl.) *181*: 458–466.

Barbaccia, M.L., Colombo, G., Affricano, D., Carai, M. a. M., Vacca, G., Melis, S., et al. (2002). GABA(B) receptor-mediated increase of neurosteroids by gamma-hydroxybutyric acid. Neuropharmacology *42*: 782–791.

Carai, M.A., Colombo, G., Brunetti, G., Melis, S., Serra, S., Vacca, G., et al. (2001). Role of GABA(B) receptors in the sedative/hypnotic effect of gamma-hydroxybutyric acid. Eur. J. Pharmacol. *428*: 315–321.

Carai, M.A.M., Agabio, R., Lobina, C., Reali, R., Vacca, G., Colombo, G., et al. (2002a). GABA(B)-receptor mediation of the inhibitory effect of gamma-hydroxybutyric acid on intestinal motility in mice. Life Sci. *70*: 3059–3067.

Carai, M.A.M., Colombo, G., and Gessa, G.L. (2005). Resuscitative effect of a gamma-aminobutyric acid B receptor antagonist on gamma-hydroxybutyric acid mortality in mice. Ann. Emerg. Med. *45*: 614–619.

Carai, M.A.M., Colombo, G., Reali, R., Serra, S., Mocci, I., Castelli, M.P., et al. (2002b). Central effects of 1,4-butanediol are mediated by GABAB receptors via its conversion into γ-hydroxybutyric acid. Eur. J. Pharmacol. *441*: 157–163.

Carter, L.P., Flores, L.R., Wu, H., Chen, W., Unzeitig, A.W., Coop, A., et al. (2003). The role of GABAB receptors in the discriminative stimulus effects of gamma-hydroxybutyrate in rats: time course and antagonism studies. J. Pharmacol. Exp. Ther. *305*: 668–674.

Carter, L.P., Wu, H., Chen, W., Cruz, C.M., Lamb, R.J., Koek, W., et al. (2004). Effects of γ-Hydroxybutyrate (GHB) on Schedule-Controlled Responding in Rats: Role of GHB and GABAB Receptors. J. Pharmacol. Exp. Ther. *308*: 182–188.

Colombo, G., Agabio, R., Bourguignon, J., Fadda, F., Lobina, C., Maitre, M., et al. (1995). Blockade of the discriminative stimulus effects of gamma-hydroxybutyric acid (GHB) by the GHB receptor antagonist NCS-382. Physiol. Behav. *58*: 587–590.

Colombo, G., Agabio, R., Bourguignon, J.-J., Lobina, C., Loche, A., Maitre, M., et al. (1999). Reduction of Blood Ethanol Levels by the Gamma-Hydroxybutyric Acid Receptor Antagonist, NCS-382. Alcohol *17*: 93–95.

Erhardt, S., Andersson, B., Nissbrandt, H., and Engberg, G. (1998). Inhibition of firing rate and changes in the firing pattern of nigral dopamine neurons by gamma-hydroxybutyric acid (GHBA) are specifically induced by activation of GABA(B) receptors. Naunyn. Schmiedebergs Arch. Pharmacol. *357*: 611–619.

Gobaille, S., Hechler, V., Andriamampandry, C., Kemmel, V., and Maitre, M. (1999). γ-Hydroxybutyrate Modulates Synthesis and Extracellular Concentration of γ-Aminobutyric Acid in Discrete Rat Brain Regions In Vivo. J. Pharmacol. Exp. Ther. *290*: 303–309.

Gobaille, S., Schleef, C., Hechler, V., Viry, S., Aunis, D., and Maitre, M. (2002). Gamma-hydroxybutyrate increases tryptophan availability and potentiates serotonin turnover in rat brain. Life Sci. *70*: 2101–2112.

Goodwin, A.K., Froestl, W., and Weerts, E.M. (2005). Involvement of gamma-hydroxybutyrate (GHB) and GABA-B receptors in the acute behavioral effects of GHB in baboons. Psychopharmacology (Berl.) *180*: 342–351.

Gupta, M., Greven, R., Jansen, E.E.W., Jakobs, C., Hogema, B.M., Froestl, W., et al. (2002). Therapeutic intervention in mice deficient for succinate semialdehyde dehydrogenase (gamma-hydroxybutyric aciduria). J. Pharmacol. Exp. Ther. *302*: 180–187.

Hicks, A.R., Kapusta, D.R., and Varner, K.J. (2004). Mechanisms underlying the sympathomimetic cardiovascular responses elicited by gamma-hydroxybutyrate. J. Cardiovasc. Pharmacol. *44*: 631–638.

Koek, W., Carter, L.P., Lamb, R.J., Chen, W., Wu, H., Coop, A., et al. (2005). Discriminative Stimulus Effects of γ-Hydroxybutyrate (GHB) in Rats Discriminating GHB from Baclofen and Diazepam. J. Pharmacol. Exp. Ther. *314*: 170–179.

Koek, W., Carter, L.P., Wu, H., Coop, A., and France, C.P. (2006). Discriminative stimulus effects of flumazenil: perceptual masking by baclofen, and lack of substitution with gamma-hydroxybutyrate and its precursors 1,4-butanediol and gamma-butyrolactone. Behav. Pharmacol. *17*: 239–247.

Lamb, R.J., Munn, J., Duiker, N.J., Coop, A., Wu, H., Koek, W., et al. (2003). Interactions of γ-hydroxy butyrate with ethanol and NCS 382. Eur. J. Pharmacol. *470*: 157–162.

Martellotta, M.C., Cossu, G., Fattore, L., Gessa, G.L., and Fratta, W. (1998). Intravenous self-administration of gamma-hydroxybutyric acid in drug-naive mice. Eur. Neuropsychopharmacol. J. Eur. Coll. Neuropsychopharmacol. *8*: 293–296.

Nava, F., Carta, G., Bortolato, M., and Gessa, G.L. (2001). gamma-Hydroxybutyric acid and baclofen decrease extracellular acetylcholine levels in the hippocampus via GABA(B) receptors. Eur. J. Pharmacol. *430*: 261–263.

Pedraza, C., García, F.B., and Navarro, J.F. (2009). Neurotoxic effects induced by gammahydroxybutyric acid (GHB) in male rats. Int. J. Neuropsychopharmacol. *12*: 1165–1177.

Poggioli, R., Vitale, G., Colombo, G., Ottani, A., and Bertolini, A. (1999). Gamma-hydroxybutyrate increases gastric emptying in rats. Life Sci. *64*: 2149–2154.

Quang, L.S., Desai, M.C., Kraner, J.C., Shannon, M.W., Woolf, A.D., and Maher, T.J. (2002). Enzyme and Receptor Antagonists for Preventing Toxicity from the Gamma-Hydroxybutyric Acid Precursor 1,4-Butanediol in CD-1 Mice. Ann. N. Y. Acad. Sci. *965*: 461–472.

Ren, X., and Mody, I. (2003). Gamma-hydroxybutyrate reduces mitogen-activated protein kinase phosphorylation via GABA B receptor activation in mouse frontal cortex and hippocampus. J. Biol. Chem. *278*: 42006–42011.

Rigamonti, A.E., and Müller, E.E. (2000). Gamma-hydroxybutyric acid and growth hormone secretion studies in rats and dogs. Alcohol Fayettev. N *20*: 293–304.

Schmidt-Mutter, C., Pain, L., Sandner, G., Gobaille, S., and Maitre, M. (1998). The anxiolytic effect of gamma-hydroxybutyrate in the elevated plus maze is reversed by the benzodiazepine receptor antagonist, flumazenil. Eur. J. Pharmacol. *342*: 21–27.

Snead III, O.C. (1996). Antiabsence seizure activity of specific GABAB and γ-hydroxybutyric acid receptor antagonists. Pharmacol. Biochem. Behav. *53*: 73–79.

Tremblay, H., Godbout, R., Girodias, V., Schmitt, M., and Bourguignon, J.J. (1998). Effects of gamma-hydroxybutyrate on ventral tegmental unit activity in the rat: considerations on rem sleep control. Sleep Res. Online SRO *1*: 152–158.

van Nieuwenhuijzen, P.S., McGregor, I.S., Chebib, M., and Hunt, G.E. (2014). Regional Fos-expression induced by γ-hydroxybutyrate (GHB): comparison with γ-butyrolactone (GBL) and effects of co-administration of the GABAB antagonist SCH 50911 and putative GHB antagonist NCS-382. Neuroscience *277*: 700–715.

Wang, L.-W., Zhou, M.-Y., Jian, H.-W., and Dai, T.-J. (2014). Relationship between gamma-hydroxybutyric acid receptors and the hypnotic and analgesic effects of emulsiﬁed inhalation anesthetics. Eur. Rev. Med. Pharmacol. Sci. *18*: 2287–2293.
