# Supplementary material for: A pharmacokinetic evaluation and metabolite identification of the GHB receptor antagonist NCS‐382 in mouse informs novel therapeutic strategies for the treatment of GHB intoxication
Source: Pharmacol Res Perspect. 2016 Oct 18;4(6):e00265. doi: 10.1002/prp2.265 (PMC5115179; doi:10.1002/prp2.265)
Supplement: Supplementary file 7 — Table S2. Characteristics of candidate NCS‐382 metabolites. [file PRP2-4-e00265-s007.docx]

**S. Table 2**. Characteristics of candidate NCS-382 metabolites.

| Biotransformation | Mass Shift | Expected m/z | Peak ID | RT (min) | Peak Area (%) | Common Product ions | Common losses* |
| --- | --- | --- | --- | --- | --- | --- | --- |
| Parent |  | 217.1 |  | 9.69 | 25 |  |  |
| Tri-oxidation | 48 | 265.1 | M15 | 15.98 | 0.9 | 117.1, 175.2 | 45.9, 43.8 |
|  |  |  | M18 | 15.57 | 0.3 | 117.1, 175.2 | 45.9, 43.9 |
| Tri-Demethylation + Dehydrogenation | -44 | 173.1 | M12 | 11.15 | 16.4 | 131.3, 155.3, 171.2, 173.2, 174.4 | 46.0, 44.0, 42.7, 41.9, 18.1 |
|  |  |  | M2 | 5.09 | 16 | 131.3, 173.2 | 41.9 |
|  |  |  | M9 | 9.69 | 6.3 | 115.2, 131.3, 155.3, 157.2, 171.2, 173.2 | 46.0, 44.0, 41.9, 17.9 |
|  |  |  | M26 | 21.42 | 5 | 116, 173.2 | 46.0, 18.1 |
|  |  |  | M27 | 21.7 | 3.6 | 173.2 | 46.0, 44.0 |
|  |  |  | M19 | 16.99 | 1.3 | 117.1, 171.2, 173.2 | 46.0, 44.0, 18.1 |
|  |  |  | M24 | 20.16 | 0.7 | 173.2 | 45.9, 44.0, 18.1 |
| Oxidation | 16 | 233.1 | M4 | 7.33 | 1 | 131.3, 171.2 | 101.9, 43.9 |
|  |  |  | M3 | 6.55 | 0.5 | 115.2, 171.2 | 100.0, 44.0 |
| Keto (Ox-2H) | 14 | 231.1 | M6 | 8.51 | 0.4 | 117.1, 129.4, 143.3, 155.3, 158.4, 171.2, 173.2 | 101.9, 100.1, 87.7, 61.9, 60.0, 44.8, 18.0 |
| Glucuronidation | 176 | 393.1 | M7 | 8.9 | 0.2 | 115.2, 155.3, 157.2, 173.2 |  |
|  |  |  | M5 | 7.87 | 0.1 | None |  |
| Glucose | 162 | 379.1 | M20 | 17.48 | 0.2 | 143.3 | 61.8, 43.9, 17.9 |
|  |  |  | M23 | 17.86 | 0.1 | 117.1, 171.2 | 100.0, 85.8, 61.8, 59.9, 45.9, 43.9, 17.9 |
| Di-Oxidation | 32 | 249.1 | M22 | 17.76 | 0.4 | 173.2 | 100.1, 43.9, 17.9 |
|  |  |  | M16 | 15.77 | 0.3 | 117.1, 155.3, 173.2 | 87.7, 85.9, 45.9, 43.9, 18.1 |
|  |  |  | M21 | 17.6 | 0.2 | 117.1, 155.3, 173.2, 175.2 | 102.0, 100.1, 73.8, 45.8, 43.9, 42.8, 18.1 |
|  |  |  | M25 | 20.38 | 0.1 |  | 60.0, 43.9 |
| Demethylation + Oxidation | 2 | 219.1 | M10 | 10.58 | 0.8 | 115.2, 129.4, 158.4, 171.2, 174.4, 175.2 | 61.7,60.8, 59.9, 43.8, 42.7 |
|  |  |  | M8 | 9.69 | 0.3 | 171.2, 173.2 | 45.9 |
|  |  |  | M11 | 11.15 | 0.3 | 171.2, 173.2, 175.2 | 45.8, 43.8 |
| Demethylation | -14 | 215.1 | M1 | 4.45 | 0.8 | 116, 130.1, 157.2 | 87.1, 46.0, 44.0 |
| Dehydrogenation | -2 | 215.1 | M13 | 11.47 | 3.2 | 171.2 | 44.0 |
|  |  |  | M14 | 13.82 | 0.5 | 171.2 | 44.0 |
|  |  |  | M17 | 15.88 | 0.2 | 116, 131.3, 143.3, 155.3, 157.2, 171.2, 173.2 | 100.1, 87.8, 59.9, 45.8, 43.9, 41.9, 17.9 |
| *min. relative intensity of 5% shown. | | | | | | | |
